# Therapeutic Potential of Salviae Miltiorrhizae Radix et Rhizoma against Human Diseases Based on Activation of Nrf2-Mediated Antioxidant Defense System: Bioactive Constituents and Mechanism of Action

**DOI:** 10.1155/2018/7309073

**Published:** 2018-06-27

**Authors:** Guo-Hui Li, Yan-Ru Li, Ping Jiao, Yu Zhao, Hui-Xin Hu, Hong-Xiang Lou, Tao Shen

**Affiliations:** ^1^Department of Pharmacy, Jinan Maternity and Child Care Hospital, Jinan, China; ^2^Key Laboratory of Chemical Biology (MOE), School of Pharmaceutical Sciences, Shandong University, Jinan, China

## Abstract

Oxidative stress plays a central role in the pathogenesis of many human diseases. The nuclear factor erythroid 2-related factor 2 (Nrf2) is a key transcription factor regulating the intracellular antioxidant response and is an emerging target for the prevention and therapy of oxidative stress-related diseases. Salviae Miltiorrhizae Radix et Rhizoma (SMRR) is a traditional Chinese medicine (TCM) and is commonly used for the therapy of cardiac cerebral diseases. Cumulative evidences indicated that the extract of SMRR and its constituents, represented by lipophilic diterpenoid quinones and hydrophilic phenolic acids, were capable of activating Nrf2 and inhibiting oxidative stress. These bioactive constituents demonstrated a therapeutic potential against human diseases, exemplified by cardiovascular diseases, neurodegenerative diseases, diabetes, nephropathy, and inflammation, based on the induction of Nrf2-mediated antioxidant response and the inhibition of oxidative stress. In the present review, we introduced the SMRR and Nrf2 signaling pathway, summarized the constituents with an Nrf2-inducing effect isolated from SMRR, and discussed the molecular mechanism and pharmacological functions of the SMRR extract and its constituents.

## 1. Introduction

Oxidative stress is defined as an imbalance of the oxidants/antioxidants tilting toward an oxidative status and characterized by the overproductions of reactive oxygen species (ROS) and reactive nitrogen species (RNS) compared with the basal state [1]. Cumulative evidences have verified that oxidative stress impairs cellular components (e.g., lipids, proteins, and nucleic acids) and plays a central role in the pathogenesis of many human diseases, such as cardiovascular diseases, neurodegenerative diseases, chronic obstructive pulmonary disease (COPD), atherosclerosis, chronic kidney diseases, diabetes, and cancer [2–8]. To eliminate excess oxidants and maintain intracellular redox homeostasis, cells have developed an adaptive and dynamic antioxidant defense system, including antioxidant molecules, antioxidant enzymes, and phase II detoxifying enzymes, to protect cells and tissues against oxidative insults.

The nuclear factor erythroid 2-related factor 2 (Nrf2) is a key transcription factor regulating the intracellular antioxidant response and plays a vital role in maintaining intracellular redox homeostasis [9]. The predominant biological function of Nrf2 is to activate the transcriptions of a wide array of cytoprotective genes that are capable of counteracting the harmful effects caused by oxidative stress and toxicants. Activation of the Nrf2-mediated cellular defense system definitely intervenes the pathogenesis of oxidative stress-induced diseases, such as cancer [10], diabetes [11], respiratory diseases [12], chronic inflammation [13], cardiovascular diseases [14], and neurodegenerative diseases [15]. The protective roles of Nrf2 against oxidative insults and xenobiotic have also been verified by bioassays using Nrf2-null mice. For example, Nrf2-null mice are more susceptible to cigarette smoke-induced emphysema [16], acetaminophen-induced hepatotoxicity [17], and benzo[a]pyrene-induced carcinogenesis [18]. Therefore, the activation of the Nrf2-mediated antioxidant defense system is an efficient strategy for the prevention and therapy of these diseases.

Natural product without a doubt is an invaluable source for discovering Nrf2 activators, and nowadays, plenty of natural molecules demonstrate therapeutic potentials against oxidative stress-related disease due to their functions on the activation of Nrf2 [19]. In our systematic investigation of Nrf2 activators from traditional Chinese medicines (TCM) [1, 20–22], we found that the extract of Salviae Miltiorrhizae Radix et Rhizoma (SMRR) promoted the activity of Nrf2-mediated phase II detoxifying enzyme, NAD(P)H: quinone reductase, and thus displayed potency on the activation of the Nrf2 signaling pathway [21]. Consistent with our observations, some literatures indicated that lipophilic diterpenoid quinones and hydrophilic phenolic acids, two types of predominant bioactive ingredients of SMRR, possessed evident Nrf2-inducing properties and inhibited the pathogenesis of diseases, exemplified by Alzheimer's disease [23], cardiovascular diseases [24, 25], and hepatic injury [26].

Although some reviews concerning the chemistry and pharmacology of SMRR have been published [27, 28], chemical constituents with Nrf2-inducing effects and their pharmacological functions based on the activation of Nrf2 have not been summarized. In this review, we introduced the SMRR and Nrf2 pathway, summarized the Nrf2 activators from SMRR, and discussed their molecular mechanisms and pharmacological functions against human diseases.

## 2. Nrf2 Signaling Pathway

**Figure 1:**
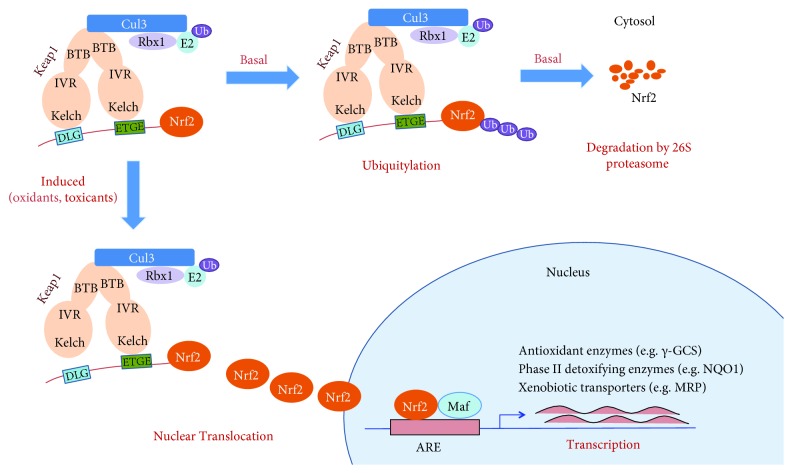
Schematic model of the regulation of the Nrf2 signaling pathway. Under basal conditions, Nrf2 undergoes Keap1-mediated ubiquitylation and 26S proteasome-mediated degradation. In response to oxidants, toxicants, or Nrf2 inducers, Nrf2 is released from Keap1, translocates into the nucleus, and activates the transcription of ARE-mediated protective genes.

Nrf2 is a basic leucine zipper (bZIP) transcription factor bearing a Cap‘n'collar (CNC) structure [29]. It possesses seven functional domains, named as Neh1–Neh7. The Neh2 domain is the key regulatory domain with two binding sites (termed as ETGE and DLG motifs) that interact with its negative regulator, Kelch-like ECH-associated protein 1 (Keap1), a substrate adaptor protein for the cullin 3- (Cul3-) containing E3 ubiquitin ligase (Figure 1) [30, 31]. Keap1 contains three functional domains that are the broad complex/tramtrack/bric-a-brac (BTB) domain, intervening region (IVR), and Kelch domain. The N-terminal BTB domain is involved in the dimerization of Keap1 via binding with Cul3. The Kelch domain interacts with the Neh2 domain of Nrf2 and regulates its physiological functions.

Under a normal state, Nrf2 is primarily localized in a complex with its repressor Keap1 via direct protein-protein interactions and is maintained at a low level in cytosol through Keap1-mediated ubiquitylation and 26S proteasome-mediated degradation. Upon exposure of cells to oxidative stress, Nrf2 is released from Keap1, translocates into the nucleus, binds to the antioxidant response element (ARE) located in the promoter region of cytoprotective genes, and activates their transcription. These ARE-containing genes are mainly divided into three groups: (i) intracellular redox-balancing proteins, including *γ*-glutamyl cysteine synthetase (*γ*-GCS), thioredoxin reductase (TrxR), and heme oxygenase-1 (HO-1) that maintain the cellular redox capacity and eliminate ROS; (ii) phase II detoxifying enzymes, exemplified by NAD(P)H: quinone oxidoreductase 1 (NQO1) and glutathione S-transferase (GST), which promote excretion of toxicants; and (iii) xenobiotic transporters: multidrug resistance-associated protein (MRP) [9, 32]. The schematic model of the Nrf2 pathway is shown in Figure 1. Based on the biological functions of these genes, the activation of Nrf2-mediated defensive response efficiently counteracts oxidative insults.

## 3. Salviae Miltiorrhizae Radix et Rhizoma (SMRR)

**Figure 2:**
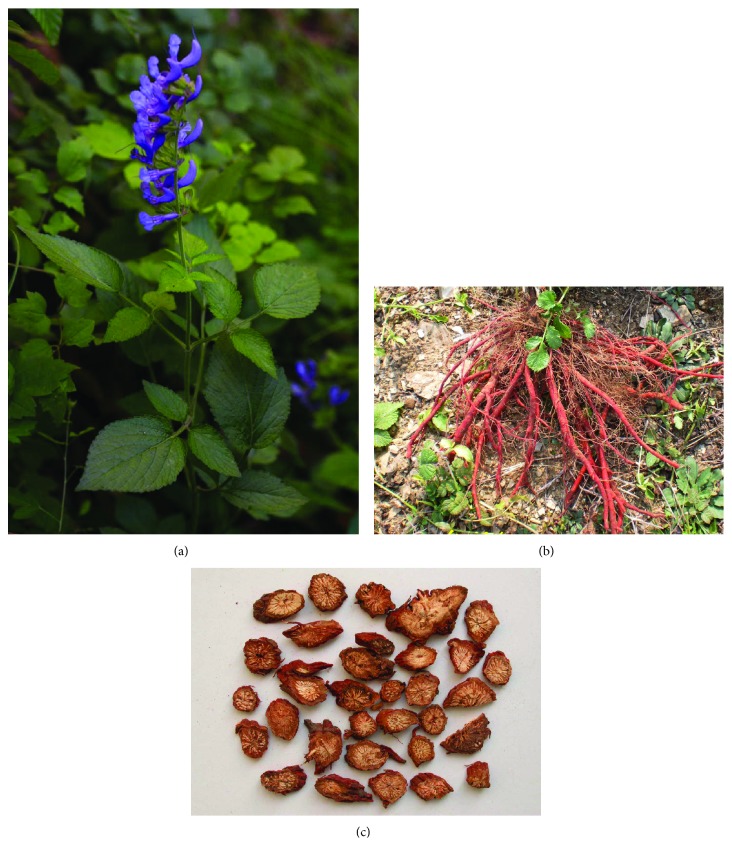
Pictures of *Salvia miltiorrhiza* and Salviae Miltiorrhizae Radix et Rhizoma: (a) the whole plant of *S. miltiorrhiza*; (b) the roots of *S. miltiorrhiza*; and (c) medicinal materials of SMRR used in TCM.

Salviae Miltiorrhizae Radix et Rhizoma, named as “Danshen” in Chinese, is the roots and rhizomes of *Salvia miltiorrhiza* Bunge (Labiatae) (Figure 2) and has a long history for the therapy of human diseases in China. Its medical applications have been recorded in many Chinese medical documents, including “Shennong's Classic of Materia Medica” (Shennong Ben Cao Jing) and “Compendium of Materia Medica” (Ben Cao Gang Mu). According to the theory of TCM, it possesses the capabilities of promoting blood flow in menstruation, removing blood stasis, relieving pain, resolving mental uneasiness and restlessness, nourishing the blood, and tranquilizing the mind (Chinese Pharmacopoeia, 2015). Presently, it is predominantly used in multicomponent preparations (e.g., Fufang Danshen Tablets, Fufang Danshen Dripping Pills, and Guanxin Danshen Capsules) to treat cardiac cerebral diseases (e.g., ischemic heart disease, ischemic stroke, coronary heart disease, angina, and thrombosis) owing to SMRR's biological functions of inhibiting platelet aggregation, modulating endothelial cell permeability, and protecting cells from ischemia-reperfusion (I/R) injury. Besides these pharmacological effects, a growing body of data indicated that SMRR potently activated intracellular antioxidant enzymes, upregulated endogenous antioxidants, and scavenged intracellular ROS. Therefore, SMRR demonstrated potential therapeutic effects against oxidative stress-induced diseases, such as cardiovascular diseases, inflammation, and renal disease [33–35].

## 4. Chemical Ingredients from SMRR and Their Potential Nrf2-Inducing Effects

**Figure 3:**
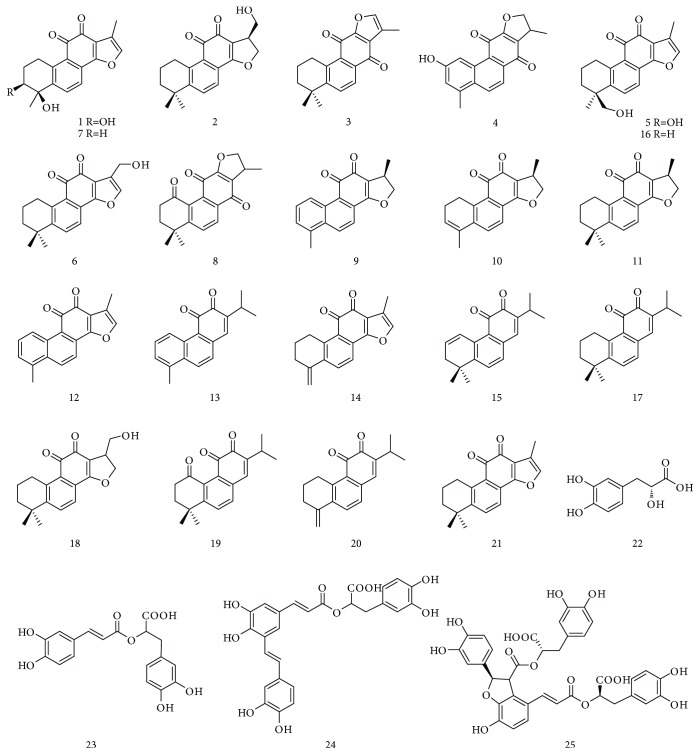
Chemical constituents with potential Nrf2-inducing effects isolated from SMRR.

Since the long history of traditional applications and significant therapeutic effects against human diseases, a large number of phytochemical investigations of SMRR have been performed to give the isolation of diverse chemical ingredients. The structural information of the purified ingredients has been summarized by some reviews [27, 36, 37]. Based on their structural characteristics and molecular polarity, SMRR-derived chemical ingredients are classified into two groups: (i) lipophilic diterpenoid quinones, which are commonly abietane-type diterpenoid skeleton with quinone moiety, including tanshinone I (**12**), tanshinone IIA (**16**), and miltirone (**17**), and (ii) hydrophilic phenolic acids that are condensed caffeic acid derivatives with different linkage and degree of polymerization, such as danshensu (**22**) and salvianolic acids A-B (**24**-**25**). Up to now, more than fifty diterpenoid quinones and forty phenolic acids have been reported from SMRR [27]. Among them, tanshinone IIA (**16**) and salvianolic acid B (**25**) are considered to be biologically active substances of SMRR and thus are selected as the markers for the quality control of SMRR (Chinese Pharmacopoeia, 2015). In this section, we have only summarized the chemical ingredients with Nrf2-inducing activity. As depicted in Table 1 and Figure 3, diterpenoid quinones are the predominant Nrf2 inducers of SMRR. For instance, Zhang et al. identified twenty diterpenoid quinones, including constituents 1–17, using a strategy combining a HPLC-based high-resolution peak fractionation approach and an ARE luciferase assay in HEK 293 T cells [38]. Furthermore, diterpenoid quinones 1, 5, 9, 11, and 15–21 were verified to be potential Nrf2 inducers by a HPLC/MS/MS method combined with QR-inducing assay in Hepa 1c1c7 cells [39]. Of which, 15,16-dihydrotanshinone I (**9**), tanshinone I (**12**), tanshinone IIA (**16**), and miltirone (**17**) have been extensively investigated. The phenolic acids, including danshensu (**22**), rosmarinic acid (**23**), and salvianolic acids A-B (**24**-**25**), are capable of activating Nrf2-mediated cytoprotective responses.

## 5. Nrf2-Based Therapeutic Potential of SMRR and Its Constituents against Human Diseases

Plenty of reviews have summarized the pharmacological functions and therapeutic effects of SMRR and its constituents on cardiovascular diseases [25, 55–57], neurodegenerative disease [23], cancer [58], osteoporosis [59], diabetes, and liver fibrosis [28]. In this section, we focused on their therapeutic effects related to the activation of Nrf2-mediated antioxidant response and inhibition of oxidative stress.

### 5.1. Cardiovascular Diseases

Oxidative stress plays an important role in the pathogenesis of cardiovascular diseases, including hypertension, I/R injury, atherosclerosis, and heart failure [7, 60–62]. Inductions of the Nrf2-regulated enzymes [e.g., superoxide dismutase (SOD), HO-1, catalase (CAT), and glutathione peroxidase (GPx)] are beneficial for the therapy of these cardiovascular diseases. The aqueous extract of SMRR prevented myocardium oxidative injury in an I/R rat model [34]. It enhanced capacities of antioxidant enzymes (e.g., SOD, CAT, and GPx) and prevented myocardium cell apoptosis. Similarly, the ethanol extract of SMRR, together with tanshinone IIA (**16**) and salvianolic acid B (**25**), dose-dependently reduced the levels of myocardium malondialdehyde (MDA) and ROS and enhanced myocardium glutathione (GSH) levels in the I/R rat model [63].

Tanshinone IIA (**16**) induced the Nrf2 signaling pathway via activating ERK and PKB, as well as downregulating the levels of tumor necrosis factor (TNF-*α*) and angiotensin II, and inhibited H_2_O_2_-induced ROS production in human aortic smooth muscle cells [45]. It reversed TNF-*α*-induced downregulations of intracellular GSH, NADPH, and glucose 6-phosphate dehydrogenase (G6PDH). Tanshinone IIA (**16**) also increased the expressions of Nrf2-mediated proteins, HO-1, ATP-binding cassette transporter A1 (ABCA1), and ATP-binding cassette transporter G1 (ABCG1) in lipid-laden macrophages, and thus suppressed accumulation of cholesterol in human macrophages [64]. Tanshinone IIA (**16**) increased Nrf2 and HO-1 expressions through the activation of phosphoinositide 3-kinase (PI3K)/Akt in human umbilical vein endothelial cells (HUVECs) and protected cells against H_2_O_2_-induced HUVEC oxidative injury [65–67]. It also upregulated the GPx activity and protected J774 macrophages against H_2_O_2_-induced cell death [68]. Tanshinone IIA (**16**) attenuated cardiac dysfunction and fibrosis and prevented cardiac remodeling via downregulating NAD(P)H oxidase-derived ROS production in 2K2C hypertensive rats [69]. In addition, tanshinone IIA (**16**) inhibited the productions of oxidized low-density lipoprotein (ox-LDL) and superoxide anion and reduced MDA level in the excessive vitamin D2 and high-cholesterol diet-induced rat atherosclerotic calcification model [70].

Ox-LDL induces endothelial dysfunction and changes of intercellular adhesion molecule-1 (ICAM-1), vascular cell adhesion molecule-1 (VCAM-1), and E-selectin, which are involved in the pathogenesis of atherosclerosis. Cryptotanshinone (**11**) inhibited ox-LDL-induced membrane expressions of ICAM-1, VCAM-1, and E-selectin, which was associated with its capability of inhibiting ROS production and nuclear factor-*κ*B (NF-*κ*B) activation [71]. Miltirone (**17**) enhanced the expressions of Nrf2, HO-1, and NQO1 in human EA.hy926 endothelial cells [47]. It protected EA.hy926 cells against ox-LDL-induced endothelial insults via inhibiting ROS synthesis and upregulating SOD and glutathione S-transferase (GST) in an Nrf2/HO-1 dependent manner.

Danshensu (**22**) was investigated for its cardioprotective activity using isolated rat hearts of the I/R model [48]. It attenuated I/R injury through scavenging ROS and inhibiting the oxidative stress. A further study indicated that danshensu (**22**) upregulated endogenous antioxidant enzymes (e.g., SOD, CAT, MDA, GPx, and HO-1), which was associated with the activation of the Nrf2/Akt/ERK1/2 signaling pathway. Salvianolic acid A (**24**) inhibited the productions of intracellular ROS and MDA and alleviated the change of mitochondrial membrane potential (MMP), in the *tert*-butyl hydroperoxide- (*t*-BHP-) induced HUVEC oxidative injury model [72].

### 5.2. Neurodegenerative Diseases

Oxidative stress and its impairment to the mitochondria play a dominant role in the onset and progression of multiple neurodegenerative disorders, including Alzheimer's disease, Parkinson's disease, and Huntington's disease [73]. Based on its function on inhibiting oxidative stress, Nrf2 is considered to be an emerging target for the treatment of neurodegenerative disorders [73, 74]. Tanshinone I (**12**) activated the expressions of Nrf2, HO-1, glutamate-cysteine ligase catalytic subunit (GCLC), and glutathione cysteine ligase modulatory subunit (GLCM) in SH-SY5Y cells [42]. Tanshinone I (**12**) inhibited 6-hydroxydopamine- (6-OHDA-) induced cell death and ROS production in SH-SY5Y neuroblastoma cells [42]. In an *in vivo* assay, tanshinone I (**12**) attenuated 6-OHDA-induced striatal oxidative stress and blocked dopaminergic neurotoxicity in 6-OHDA-lesion mice. It also protected the mitochondria against paraquat-induced redox impairment via upregulating Nrf2-regulated antioxidant enzymes, such as Mn-superoxide dismutase (Mn-SOD), GPx, and *γ*-glutamate-cysteine ligase (*γ*-GCL) [43]. Similarly, tanshinone IIA (**16**) induced the expressions of Nrf2, HO-1, GCLC, and GCLM in SH-SY5Y cells and protected cells against 6-OHDA-induced ROS production and cell death in an Nrf2-dependent manner *in vitro* [46]. It ameliorated neurodegeneration in a 6-OHDA-induced rat model of Parkinson's disease.

Salvianolic acid A (**24**) enhanced neuronal survival and stabilized MMP in SH-SY5Y cells and protected SH-SY5Y cells against H_2_O_2_-induced oxidative injury [75]. This protective effect was caused by the inhibition of the AMP-activated protein kinase (AMPK) and the Akt signaling pathway. Salvianolic acid B (**25**) was evaluated for the protection against cognitive decline using a high-fat diet-fed mouse model [76]. It upregulated antioxidant enzymes (e.g. SOD and GPx), attenuated hippocampal redox status, and thus counteracted cognitive decline [76]. Salvianolic acid B (**25**) was also investigated for its neuroprotection using lipopolysaccharide- (LPS-) and 1-methyl-4-phenylpyridinium- (MPP^+^-) induced neuronal injury model *in vitro* [53]. Salvianolic acid B (**25**) upregulated Nrf2 expression and decreased LPS- and MPP^+^-induced toxicities of dopamine neurons in the primary neuroglia of a mouse. Salvianolic acid B (**25**) attenuated dopaminergic neuronal loss, inhibited neuroinflammation, and improved the neurological function of neurotoxin 1-methyl-4-phenyl-1,2,3,6-tetrahydropyridine- (MPTP-) treated mice [53].

### 5.3. Diabetes

Oxidative stress triggers the mitochondrial damage which is the predominant contributing factor of excessive *β*-cell death [77]. Furthermore, high glucose-induced redox imbalance provokes oxidative insults of human tissues and organs (e.g., cardiovascular system, kidney, and eyes) [78]. The activation of Nrf2-regulated antioxidant response relieved the pathogenesis and progression of diabetes [79, 80]. Salvianolic acid B (**25**) inhibited the development of diabetes-related nephropathy and vascular complications in a type 2 diabetic animal model [81]. It also protected pancreatic *β*-cells against cytokines, interferon-*γ* (INF-*γ*), and interleukin- (IL-) 1*β* and induced INS-1 cell death through activating the expressions of Nrf2, HO-1, and Sirt1 [82]. Salvianolic acid A (**24**) reduced the levels of advanced glycation end products (AGEs) and MDA and improved intestinal motility in diabetic rats [83].

### 5.4. Nephropathy

Oxidative stress has been implicated in the onset and promotion of nephropathy, and the modulation of Nrf2 is an efficient strategy for the therapy of renal diseases [84]. Ethyl acetate extract of SMRR enhanced the expression of Nrf2 and inhibited ROS production in high-glucose-induced mouse mesangial cells (MMCs) [33]. It reduced albuminuria and alleviated renal damage in streptozocin-induced mice. SMRR injection inhibited N(G)-nitro-d-arginine-induced oxidative injury in a rat kidney [85]. It upregulated the activities of endogenous antioxidant enzymes (e.g., SOD and GPx) and decreased the level of MDA. Salvianolic acid B (**25**) activated Nrf2, reduced cellular ROS level in HK-2 cells, and protected cells against H_2_O_2_-induced cell death [86]. Furthermore, an *in vivo* study indicated that it activated Nrf2 expression, inhibited renal oxidative stress, and attenuated renal tubular injury in iodinated contrast media-induced acute renal injury in rats. This protection against renal damage was associated with the activation of the PI3K/Akt/Nrf2 pathway.

### 5.5. Inflammation

Oxidative stress activates redox-sensitive NF-*κ*B and subsequently triggers the overproductions of proinflammatory cytokines and enzymes, such as tumor necrosis factor-*α* (TNF-*α*), ILs, cyclooxygenase-2 (COX-2), and inducible NO synthesis (iNOS) [87]. Nrf2 negatively regulated these proinflammatory cytokines and enzymes and thus inhibited inflammatory response. SMRR upregulated the GSH level and inhibited MDA level in the synovium and articular cartilage of the rabbits and prevented articular cartilage degeneration in rabbits with osteoarthritis [35]. Extract of SMRR induced the expressions of HO-1 and Nrf2 and inhibited H_2_O_2_-stimulated production of ROS in RAW 264.7 macrophages [88]. The activation of Nrf2 was attributed to the PI3K/Akt and MEK1 signaling pathway. Tanshinone IIA (**16**) induced HO-1 expression in RAW 264.7 macrophages and inhibited LPS-stimulated upregulation of COX-2 and iNOS [89]. A high-fat diet gave rise to the decrease of Nrf2 expression, accumulation of oxidative stress, and inflammation in C57BL/6 mouse. Salvianolic acid B (**25**) upregulated the expressions of Nrf2, HO-1, and NQO1 and thus inhibited the expressions of NF-*κ*B, COX-2, and iNOS in mice fed a high-fat diet [90].

### 5.6. Liver Diseases

As the major metabolism organ of xenobiotics, the liver is apt to be kept in a sustained oxidative state, which causes a variety of liver diseases, covering fibrosis, cirrhosis, and carcinoma [91, 92]. The extract of SMRR inhibited aflatoxin B1- (AFB_1_-) induced cytotoxicity in cultured primary rat hepatocytes since its capability of inhibiting oxidative stress [93]. AFB_1_-induced ROS formation and GSH depletion could be reverted by SMRR extract treatment. Tanshinone IIA (**16**) upregulated the protein level of Nrf2 and enhanced the mRNA levels of GCLC, NQO1, and HO-1 in HepG2 cells and in a C57BL/6J mouse liver [26]. Tanshinone IIA (**16**) alleviated acetaminophen- (APAP-) induced upregulations of serum alanine aminotransferase (ALT), aspartate aminotransferase (AST), and lactate dehydrogenase (LDH). It reverted the APAP-induced decreases of GSH, GST, GPx, SOD, and CAT in a mouse liver and thus prevented APAP-induced hepatotoxicity. Similarly, tanshinone IIA (**16**) upregulated the levels of Nrf2-mediated antioxidant enzymes (e.g., HO-1, SOD, CAT, and GPx) and inhibited fibrosis in a rat model of cirrhosis.

Salvianolic acid A (**24**) evidently reduced oxidative stress, evidenced by decreasing the levels of ROS and MDA and increasing the levels of hepatic superoxide dismutase and GSH, in rat liver tissue [94]. Furthermore, salvianolic acid A (**24**) significantly inhibited carbon tetrachloride- (CCl_4_-) induced hepatotoxicity. Decreased levels of serum ALT and AST in response to CCl_4_ exposure were recovered after treatment with **24**. Salvianolic acid A (**24**) demonstrated radioprotective effects against *γ*-radiation-induced damage in human embryo liver L-02 cells, which was associated with inhibitions of ROS generation and mitochondrial cytochrome C release [95]. Salvianolic acid B (**25**) demonstrated APAP-induced liver injury in mice [54]. It upregulated the expressions of Nrf2, GCLC, and HO-1 via the activation of the PI3K and protein kinase C (PKC) pathway in HepG2 cells.

### 5.7. Lung Diseases

The lung is directly and continuously exposed to the external oxidants and toxicants, and thus excessive ROS are produced in the lung tissue. These endogenous and exogenous oxidants contribute to the pathophysiology of lung diseases, exemplified by chronic obstructive pulmonary disease and pulmonary fibrosis [96, 97]. Tanshinone I (**12**) enhanced Nrf2-mediated expressions of NQO1 and *γ*-GCS through hindering Nrf2 ubiquitination and specifically reacting with cysteine residue at amino acid 151 in Keap1 protein [40]. It protected human bronchial epithelial HBE cells against As(III)-induced oxidative damage in an Nrf2-dependent manner. An *in vivo* study using Nrf2^+/+^ and Nrf2^−/−^ mice indicated that tanshinone I (**12**) attenuated As(III)-induced inflammatory lung damage in Nrf2^+/+^ mice, and this protective effect vanished in Nrf2^−/−^ mice.

Salvianolic acid B (**25**) upregulated the expressions of Nrf2 and HO-1, enhanced GSH production, and inhibited cigarette smoke-induced lung pathological changes and inflammatory responses [52]. Salvianolic acid B (**25**) reduced the expression of ROS-generating enzyme NADPH oxidase-4 (Nox4) in lung tissue of mice [98]. It inhibited paraquat-induced structure distortion, collagen overproduction, proinflammatory cytokine release, and oxidative insults. Salvianolic acid B (**25**) inhibited PQ-induced activation of the transforming growth factor-*β* (TGF-*β*1)/Smad3 pathway, which was a key regulator of pulmonary fibrosis [99]. The capability of **25** in activating Nrf2 and inhibiting pulmonary fibrosis was further confirmed by a MRC-5 human lung fibroblast cell model and a bleomycin-induced rat pulmonary fibrosis model [100].

### 5.8. Ocular Diseases

Oxidative stress is involved in the pathogenesis of age-related macular degeneration [101]. Salvianolic acid A (**24**) inhibited H_2_O_2_-induced ROS production and prevented H_2_O_2_-induced primary and transformed retinal pigment epithelial cell death and apoptosis [51]. Activations of Nrf2 and HO-1 by salvianolic acid A (**24**) are essential for this protective effect. The Nrf2-inducing effect of **24** is associated with the activations of PI3K and the mammalian target of rapamycin (mTOR) signaling pathway [50]. It attenuated ox-LDL-induced lipidosis and apoptosis in the retinal pigment epithelium layer and inhibited ox-LDL-induced elevated ROS level and RPE inflammation in a rat model. These data suggested that salvianolic acid A (**24**) was a potential therapeutic agent against age-related macular degeneration.

### 5.9. Others

Because of wide distribution in the human tissues and organs, the activation of Nrf2 is an effective method for the therapy of many human diseases [102, 103]. Noise-induced hearing loss is associated with oxidative stress and lipoperoxidative damage. Rosmarinic acid (**23**) enhanced Nrf2/HO-1-mediated endogenous antioxidant defense system, attenuated hearing loss, and promoted hair cell survival in a noise-induced rat model [49]. Tanshinone I (**12**) and 15,16-dihydrotanshinone (**9**) induced the expressions of Nrf2, GCLC, and NQO1 through inhibiting Nrf2 ubiquitination in human Hs27 dermal fibroblasts and HaCaT keratinocytes [41]. These two constituents significantly suppressed solar-simulated UV and riboflavin-sensitized ultraviolet-induced skin cell death. A solar-simulated UV-induced human skin reconstruct model was established for evaluating the photoprotective effect of 15,16-dihydrotanshinone (**9**). Treatment with 15,16-dihydrotanshinone (**9**) definitely attenuated epidermal solar insult.

## 6. Conclusion and Future Perspectives

Oxidative stress plays a vital role in the pathogenesis of many human diseases. The activation of the Nrf2-mediated antioxidant defense system inhibits oxidative stress and thus effectively blocks the onset and progression of oxidative stress-induced human diseases. SMRR is a traditional Chinese medicine that has been long used for the treatment of cardiac cerebral diseases. Lipophilic diterpenoid quinones and hydrophilic phenolic acids are predominant constituents and contribute to the pharmacological functions of SMRR. These two types of constituents demonstrate potent Nrf2-inducing effects and hence are potential agents for the treatment of oxidative stress-related diseases. The SMRR extract, as well as the purified constituents, tanshinone I (**12**), tanshinone IIA (**16**), salvianolic acids A (**24**), and B (**25**), has been extensively investigated, and their inductions on the Nrf2 pathway and therapeutic effects on cardiovascular diseases, neurodegenerative diseases, diabetes, nephropathy, inflammation, liver diseases, and lung diseases have been verified by multiple models *in vitro* and *in vivo*.

Although there has been great progress on SMRR and its constituents, important areas on their phytochemistry, pharmacology, and medical applications related to the activation of Nrf2 remain to be explored. (i) Plenty of diterpenoid quinones have been isolated from SMRR; however, extensive researches still focused on several active constituents, including 15,16-dihydrotanshinone I (**9**), tanshinone I (**12**), tanshinone IIA (**16**), and miltirone (**17**). Miscellaneous diterpenoid quinones should be extensively investigated, such as their structure-activity relationship and mechanisms of action on the activation of Nrf2. (ii) Besides these verified medical applications, SMRR and its constituents possess potential pharmacological functions (e.g., chemoprevention on cancer and therapeutic effect on diabetic nephropathy) because of their activation of Nrf2. Thus, these unconfirmed pharmacological activities should be noted. (iii) Based on the traditional uses, chemical constituents, and pharmacological functions of SMRR, future works on discovering new lead compounds and developing SMRR and its constituents into new drugs for the therapy of oxidative stress-related diseases are significant.

## Figures and Tables

**Table 1:** Chemical constituents with Nrf2-inducing activity.

Structural type	**No**	Name of compounds	Nrf2-inducing activity	Cell or animal models	Ref
Diterpenoid quinones	**1**	Tanshindiol B	6.80-fold induction of ARE luciferase activity	HEK 293 T cells	[38]
			4.85 *μ*M for 2-fold induction of QR activity	Hepa 1c1c7 cells	[39]
	**2**	17-Hydroxycryptotanshinone	3.97-fold induction of ARE luciferase activity	HEK 293 T cells	[38]
	**3**	Isotanshinone IIA	6.45-fold induction of ARE luciferase activity	HEK 293 T cells	[38]
	**4**	Trijuganone A	4.31-fold induction of ARE luciferase activity	HEK 293 T cells	[38]
	**5**	Tanshinone IIB	5.34-fold induction of ARE luciferase activity	HEK 293 T cells	[38]
			4.85 *μ*M for 2-fold induction of QR activity	Hepa 1c1c7 cells	[39]
	**6**	Przewaquinone A	6.01-fold induction of ARE luciferase activity	HEK 293 T cells	[38]
	**7**	Przewaquinone B	7.27-fold induction of ARE luciferase activity	HEK 293 T cells	[38]
	**8**	1-Ketoisocryptotanshinone	5.65-fold induction of ARE luciferase activity	HEK 293 T cells	[38]
	**9**	15,16-Dihydrotanshinone I	3.38-fold induction of ARE luciferase activity	HEK 293 T cells	[38]
			4.80 *μ*M for 2-fold induction of QR activity	Hepa 1c1c7 cells	[39]
			Induction of ARE luciferase and upregulation of Nrf2 protein level	MDA-MB-231 cells	[40]
			Induction of Nrf2, NQO1, and *γ*-GCS protein levels	Human dermal fibroblasts.	[41]
	**10**	1,2,15,16-Tetrahydrotanshinone I	4.37-fold induction of ARE luciferase activity	HEK 293 T cells	[38]
	**11**	Cryptotanshinone	3.61-fold induction of ARE luciferase activity	HEK 293 T cells	[38]
			5.07 *μ*M for 2-fold induction of QR activity	Hepa 1c1c7 cells	[39]
			Induction of ARE luciferase and upregulation of Nrf2 protein level	MDA-MB-231 cells	[40]
			Induction of ARE-luciferase activity	Hep G2 cells	[26]
	**12**	Tanshinone I	2.60-fold induction of ARE luciferase activity	HEK 293 T cells	[38]
			5.90 *μ*M for 2-fold induction of QR activity	Hepa 1c1c7 cells	[39]
			Induction of ARE luciferase and upregulation of Nrf2 protein level	MDA-MB-231 cells	[40]
			Upregulation of Nrf2, HO-1, GCLC, and GCLM	SH-SY5Y cells	[42, 43]
			Induction of ARE-luciferase activity	Hep G2 cells	[26]
			Induction of Nrf2, NQO1, and *γ*-GCS protein levels	Human dermal fibroblasts	[41]
	**13**	RO-090680	3.06-fold induction of ARE luciferase activity	HEK 293 T cells	[38]
	**14**	Methylenetanshinquinone	2.77-fold induction of ARE luciferase activity	HEK 293 T cells	[38]
	**15**	1,2-Didehydromilitirone	3.79-fold induction of ARE luciferase activity	HEK 293 T cells	[38]
			1.04 *μ*M for 2-fold induction of QR activity	Hepa 1c1c7 cells	[39]
	**16**	Tanshinone IIA	2.58-fold induction of ARE luciferase activity	HEK 293 T cells	[38]
			5.10 *μ*M for 2-fold induction of QR activity	Hepa 1c1c7 cells	[39]
			Upregulation of Nrf2, HO-1, and NQO-1 protein levels	JB6 cells	[44]
			Induction of ARE luciferase and upregulation of Nrf2 protein level	MDA-MB-231 cells	[40]
			Upregulation of Nrf2, NOQ-1, HO-1, GCLC, and GCLM protein levels	Human aortic smooth muscle cells.	[45]
			Induction of ARE-luciferase activity and upregulation of mRNA levels of GCLC, NQO1, and HO-1	Hepa G2 cells	[26]
			Induction of Nrf2 and upregulation of mRNA and protein levels of HO-1, NQO1, and GCLC	SH-SY5Y cells	[46]
	**17**	Miltirone	3.32-fold induction of ARE luciferase activity	HEK 293 T cells	[38]
			0.92 *μ*M for 2-fold induction of QR activity	Hepa 1c1c7 cells	[39]
			Enhancement of Nrf2 translocation and upregulation mRNA and protein levels of Nrf2, HO-1, and NQO1	EA.hy926 cells	[47]
	**18**	17-Hydroxycryptotanshinone	4.85 *μ*M for 2-fold induction of QR activity	Hepa 1c1c7 cells	[39]
	**19**	1-Oxomiltirone	0.40 *μ*M for 2-fold induction of QR activity	Hepa 1c1c7 cells	[39]
	**20**	4-Methylenemiltirone	0.46 *μ*M for 2-fold induction of QR activity	Hepa 1c1c7 cells	[39]
	**21**	1,2-Dihydrotanshinone I	5.40 *μ*M for 2-fold induction of QR activity	Hepa 1c1c7 cells	[39]

Phenolic acids	**22**	Danshensu	Upregulation of mRNA and protein levels of Nrf2	Rat heart	[48]
	**23**	Rosmarinic acid	Upregulation of Nrf2 and HO-1 protein level	Rat cochlea	[49]
	**24**	Salvianolic acid A	Induction of mRNA and protein levels of Nrf2 and HO-1	RPE cells	[50, 51]
	**25**	Salvianolic acid B	Induction of Nrf2 and HO-1	Rat lung tissue	[52]
			Induction of Nrf2 protein level	Rat primary neurons	[53]
			Induction of Nrf2, HO-1, and GCLC protein levels	Hepa G2 cells	[54]

## Abbreviations

ABCA1: ATP-binding cassette transporter A1
AFB_1_: Aflatoxin B1
AGEs: Advanced glycation end products
ALT: Alanine aminotransferase
APAP: Acetaminophen
AST: Aspartate aminotransferase
ARE: Antioxidant response element
BTB: Broad complex/tramtrack/bric-a-brac
CAT: Catalase
COX-2: Cyclooxygenase-2
*γ*-GCL: *γ*-Glutamate cysteine ligase
GCLC: Glutamate cysteine ligase catalytic subunit
GCLM: Glutathione cysteine ligase modulatory subunit
*γ*-GCS: *γ*-Glutamyl cysteine synthetase
GPx: Glutathione peroxidase
GSH: Glutathione
GST: Glutathione S-transferase
HO-1: Heme oxygenase-1
ICAM-1: Intercellular adhesion molecule-1
HUVECs: Human umbilical vein endothelial cells
IL: Interleukin
INF-*γ*: Interferon-*γ*
iNOS: Inducible NO synthesis
I/R: Ischemia-reperfusion
IVR: Intervening region
Keap1: Kelch-like ECH-associated protein 1
LDH: Lactate dehydrogenase
LPS: Lipopolysaccharide
MDA: Malondialdehyde
MMP: Mitochondrial membrane potential
Mn-SOD: Mn-superoxide dismutase
MPP^+^: 1-Methyl-4-phenylpyridinium
MPTP: 1-Methyl-4-phenyl-1,2,3,6-tetrahydropyridine
mTOR: Mammalian target of rapamycin
NF-*κ*B: Nuclear factor-*κ*B
NQO1: NAD(P)H: quinone oxidoreductase 1
Nrf2: Nuclear factor erythroid 2-related factor 2
6-OHDA: 6-Hydroxydopamine
ox-LDL: Oxidized low-density lipoprotein
PKC: Protein kinase C
PI3K: Phosphoinositide 3-kinase
RNS: Reactive nitrogen species
ROS: Reactive oxygen species
SMRR: Salviae Miltiorrhizae Radix et Rhizoma
SOD: Superoxide dismutase
*t*-BHP: *tert*-Butyl hydroperoxide
TCM: Traditional Chinese medicines
TGF-*β*1: Transforming growth factor-*β*
TNF-*α*: Tumor necrosis factor-*α*
TrxR: Thioredoxin reductase
VCAM-1: Vascular cell adhesion molecule-1.
